# Patient-centred communication in primary TB care delivery in Western China

**DOI:** 10.5588/ijtldopen.26.0073

**Published:** 2026-08-11

**Authors:** J. Zhou, Q. Huang, L. Luo, T. Zhang, G. Wang, X. Zhao, S. Liu, Q. Yuan, Y. Chen, Y. Li

**Affiliations:** 1 Department of Social Medicine and Health Service Management, Army Medical University (Third Military Medical University), Shapingba, Chongqing, China;; 2 Department of TB Control, Center of Disease Control and Prevention, Guiyang, Guizhou, China;; 3 Department of Districts and Counties, Chongqing Institute of TB Prevention and Treatment, Jiulongpo, Chongqing, China;; 4 Guiyang Center for Disease Control and Prevention, Guiyang, Guizhou, China.

**Keywords:** tuberculosis, patient-centred care, 4-Habits Coding Scheme, cross-cultural adaptation

## Abstract

**BACKGROUND:**

Patient-centred communication (PCC) is critical for delivering high-quality TB care. The 4-Habits Coding Scheme (4-HCS) is a tool designed to assess 23 communication behaviours from a patient-centred perspective.

**METHODS:**

We aimed to cross-culturally adapt and validate the 4-HCS into Chinese and evaluate PCC skills among TB health care workers (HCWs) in primary health care (PHC) settings in Western China.

**RESULTS:**

The Chinese version of the 4-HCS demonstrated robust reliability, face validity, and construct validity, and construct validity, and a total of 2,807 HCWs were included in the study. Community-level TB HCWs (3.872 ± 0.842) had better PCC skills than village-level HCWs (3.748 ± 0.860). The Habit 4 (Invest in the End) dimension which required more TB treatment knowledge had the lowest scores across four dimensions at both levels. Factors associated with higher 4-HCS scores included positive working attitudes, high working satisfaction, more years of TB working experience, and being from the community level (all *P* < 0.05).

**CONCLUSION:**

The Chinese version of the 4-HCS is a reliable and valid tool for assessing PCC skills among TB HCWs in PHC settings. Future research should expand to diverse regions and incorporate objective assessments to further validate the tool and explore its broader applicability.

TB remains a major global health challenge, and one of the leading causes of death worldwide.^1^ According to the WHO Global TB Report 2025, an estimated 10.7 million people fell ill with TB, and 1.23 million died from the disease.^1^ China remains among the 30 high-TB-burden countries, accounting for 6.5% of global new TB cases.^1^ The WHO End TB Strategy identifies integrated patient-centred care as a core pillar for ending the TB epidemic by 2030.^2^ Patient-centred care emphasises individualised, holistic, and empowering approaches that promote shared decision-making and patient engagement.^3^ Given TB’s prolonged treatment course and infectious nature, patient-centred care is particularly critical for effective case management and sustained treatment adherence.^4^ Adherence to anti-TB treatment is essential for cure and for preventing drug-resistant TB.^5,6^

Under China’s integrated TB control model, TB health care workers (HCWs) in primary health care (PHC) settings play a central role in TB case management, including health education, management of adverse effects, and treatment follow-up.^7^ Evidence from China indicates that improved doctor–patient communication enhances treatment outcomes by addressing patients’ informational and emotional needs.^8,9^ Effective patient-centred communication (PCC) between TB patients and HCWs is therefore crucial for improving patient satisfaction, care quality, and treatment outcomes, as well as reducing TB transmission.^10–12^ However, few studies have systematically assessed PCC skills among TB HCWs using validated instruments. The 4-Habits Coding Scheme (4-HCS) is a standardised and validated tool designed to assess 23 communication behaviours from a patient-centred perspective.^13^ Grounded in the 4-Habits Model, it emphasises partnership-building, empathy, and shared decision-making throughout clinical encounters.^14^ The 4-HCS has been widely used and cross-culturally adapted in multiple languages, including French, German, Norwegian, and Brazilian Portuguese.^15–18^ It comprises four domains: Invest in the Beginning, Elicit the Patient’s Perspective, Demonstrate Empathy, and Invest in the End.^17^

We acknowledge the previous work by the Chinese research team that adapted the 4-HCS into the Five Habits Coding Scheme version by adding a new dimension. However, our study focuses on the translation and cultural adaptation of the original 4-HCS for use in primary care settings in China. This study therefore aimed to translate, cross-culturally adapt, and validate the original 4-HCS into Chinese. A secondary objective was to apply the Chinese version to assess PCC skills among TB HCWs in PHC settings and to explore associated factors in Western China.

## METHODS

This study utilised a two-step procedure. Firstly, the 4-HCS was translated, cross-culturally adapted, and validated into Chinese from October to November 2021. Secondly, a cross-sectional study of the cross-culturally adapted Chinese version of 4-HCS was conducted to assess PCC skills among TB HCWs in PHC settings in Western China from December 2021 to May 2022.

### Cross-cultural adaptation of the 4-HCS into Chinese

The aim of translation, cross-cultural adaptation, and validation was to obtain a Chinese version of 4-HCS that was conceptually equivalent to the original US source version, culturally relevant to the Chinese context, and user-friendly for the target HCWs who would use the scale.^19^ For this purpose, we used a rigorously methodological approach involving input from the 4-HCS developer on conceptual issues and a centralised review process coordinated by a consultant with experience in TB prevention and control in PHC settings. This approach included five steps: 1) translation of the original 4-HCS into Chinese; 2) comparison of the four translated versions of the 4-HCS; 3) blind back-translation of the preliminary initial translated version of 4-HCS; 4) comparison of the two back-translated versions of 4-HCS; and 5) pilot testing the final version of 4-HCS in Chinese.

### Study setting

**Figure 1.**
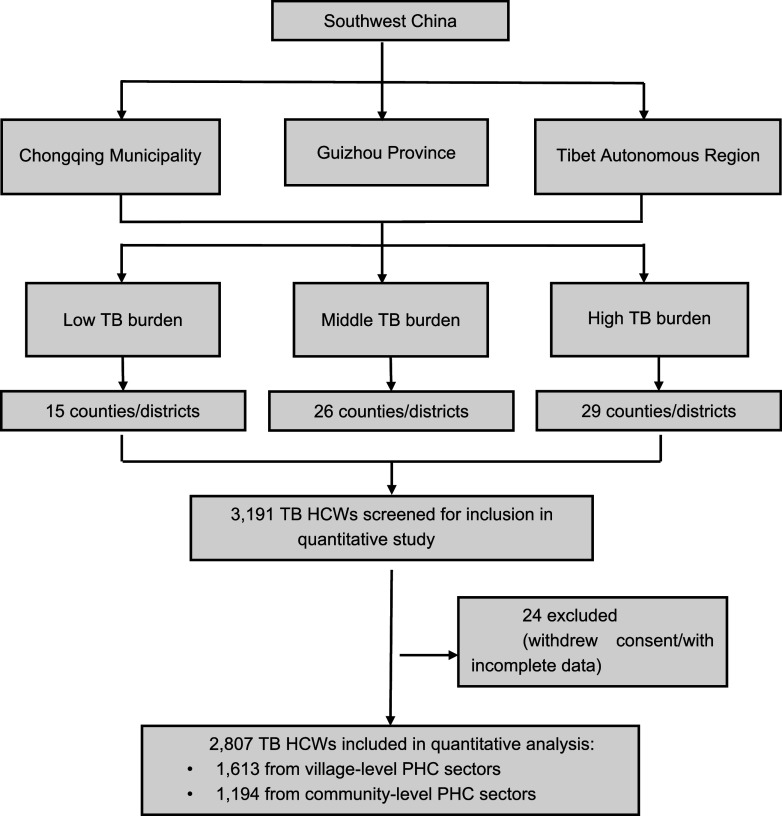
Study flow chart of mixed-study in Chongqing municipality, Guizhou province, and Tibet autonomous region. HCW = health care worker; PHC = primary health care.

A multistage stratified random sampling method was used to select PHC sectors in Chongqing Municipality, Guizhou Province, and the Tibet Autonomous Region in Western China, where TB prevalence is significantly higher than in central and eastern regions. Chongqing, Guizhou, and Tibet were purposively selected to represent areas with low, middle, and high TB burden and corresponding socio-economic status, respectively. Counties/districts within each region were stratified into low-, middle-, and high-TB-incidence groups, and at least three counties from each stratum were randomly selected. In total, 70 counties/districts were included in the study (Figure 1). The study design and reporting followed the Strengthening the Reporting of Observational Studies in Epidemiology (STROBE) guidelines for observational studies.^20^

### Study participants, sample size, and data collection

All TB HCWs in PHC sectors at community (community health centres/stations and township hospital centres) and village level (village clinics) who were responsible for TB control implementation from the selected 70 counties/districts were targeted in this survey. A structured questionnaire was developed, covering 1) demographic information, such as age and education; 2) working information, such as professional title and job satisfaction; and 3) the Chinese version of 4-HCS. Those willing to participate were recruited with the assistance of local Centers for Disease Control and Prevention. The sample size was calculated using the stratified sampling: $n=\frac{\sum W_{i}^{2}S_{i}^{2}/w_{i}}{V+\sum W_{i}S_{i}^{2}/N}$, where*, n* is the minimum desired sample size, *W*_*i*_ *= N*_*i*_*/N*, V=(δ/u_α/2_)^2^, and u_α/2_ = 1.96, corresponding to a 5% level of significance. The minimum required sample size was approximately 500 per provincial-level region, resulting in a total minimum sample of 1,500. Quality control included random review of 10% of questionnaires, yielding a compliance rate above 95%. Participation was voluntary, and informed consent was obtained prior to data collection. All completed questionnaires were checked by trained investigators.

### Data analysis

The double-checking method and SPSS 26.0 (IBM Corporation, Armonk, NY, USA) was used for data analysis. Reliability and validity analysis of the Chinese version of 4-HCS was conducted to test the validity and consistency of the questionnaire, and the Kaiser–Meyer–Olkin (KMO) and Cronbach’s α values were used to evaluate the internal consistency and face validity of the Chinese 4-HCS.^21,22^ Descriptive statistical analysis was used to investigate TB HCWs’ demographic, working characteristics, and PCC skills. Categorical variables were represented by frequencies and percentage (n, %). Mean scores and standard distribution (mean, SD) of responses for each Habit and each item of Chinese 4-HCS were calculated. The χ² test, Mann–Whitney *U* test, and Kruskal–Wallis test were utilised to process univariate analysis. Linear logistic regression was then used to assess associations between characteristics and 4-HCS responses. A two-tailed probability level of *P* < 0.05 was considered statistical significance.

### Ethical statement

The project proposal was approved by the Institutional Review Board of Army Medical University (Third Military Medical University), Chongqing, China (Grant No. 2021-03-02 and No. 2024-37-02). This study was conducted in accordance with the Declaration of Helsinki. When participants began the online questionnaire, the first page detailed the informed consent. Participants had to click the option ‘Yes, I consent’ in order to proceed to the start page of the survey. Completed informed consents were obtained from all participants.

## RESULTS

### Test of Chinese version of 4-HCS

The self-reported pre-final Chinese version of 4-HCS was pilot-tested among 89 primary HCWs in November 2021. Reliability evaluation revealed a Cronbach’s α of 0.967 (>0.8), indicating a good reliability. Sensitivity analysis showed that the coefficients after deleting each item ranged from 0.964 to 0.967, suggesting high consistency across all items. Construct validity was confirmed by a KMO coefficient of 0.902 (>0.5) and a Bartlett’s test result of 1978.895 (*P* < 0.001), demonstrating strong construct validity. Following a rigorous five-step translation and cross-cultural adaptation process, a total of 2,807 responses were collected for the final Chinese version of the 4-HCS. Reliability and validity analyses of the final version indicated a KMO coefficient of 0.986 (>0.5) and a Cronbach’s α of 0.990 (>0.8), further confirming excellent construct validity and internal consistency. In the face validity analysis, results showed that the Corrected item-total correlations ranged between 0.65 and 0.91, which indicates that the questionnaire had good face validity.

### Demographic and working characteristics of participants

A total of 2,807 TB HCWs were included in the final analysis (1,613 village-level; 1,194 community-level) (Table 1). Community-level HCWs were younger and more educated, whereas village-level HCWs were older, less educated, more likely to lack professional titles, had longer TB work experience, and undertook more basic public health care service programmes. Community-level HCWs reported higher job satisfaction (55.0% vs. 49.5%), while village-level HCWs showed greater willingness to continue TB work (74.5% vs. 65.7%) and received more frequent TB programme training (all *P* < 0.001).

**Table 1.** Demographic and working-related characteristics of TB HCWs, No. (%).

Characteristics	Village level (n = 1,613)	Community level (n = 1,194)	*P* value	Village level (n = 1,613)			*P* value	Community level (n = 1,194)			*P* value
				Chongqing (n = 710)	Guizhou (n = 739)	Tibet (n = 164)		Chongqing (n = 369)	Guizhou (n = 542)	Tibet (n = 283)	
Gender			<0.001*				0.001				<0.001*
Women	913 (56.6)	527 (44.1)		414 (58.3)	388 (52.5)	111 (67.7)		118 (32.0)	222 (41.0)	187 (66.1)	
Men	700 (43.4)	667 (55.9)		296 (41.7)	351 (47.5)	53 (32.3)		251 (68.0)	320 (59.0)	96 (33.9)	
Age, years			<0.001*				<0.001*				<0.001*
20–29	236 (16.3)	504 (42.2)		65 (9.2)	93 (12.6)	105 (64.0)		142 (38.5)	165 (30.4)	197 (69.6)	
30–39	337 (20.9)	359 (30.1)		139 (19.6)	151 (20.4)	47 (28.7)		119 (32.3)	165 (30.4)	75 (26.5)	
40–49	638 (39.6)	242 (20.3)		294 (41.4)	337 (45.6)	7 (4.3)		75 (20.3)	157 (29.0)	10 (3.5)	
>49	375 (23.2)	89 (7.5)		212 (29.9)	158 (21.4)	5 (3.0)		33 (8.9)	55 (10.1)	1 (0.4)	
Education level			<0.001*				<0.001*				<0.001*
Technical secondary school/below	829 (51.4)	158 (13.2)		410 (57.7)	379 (51.3)	40 (24.4)		52 (14.1)	96 (17.7)	50 (11.2)	
Junior medical college	589 (36.5)	544 (45.6)		237 (33.4)	284 (38.4)	68 (41.5)		175 (47.4)	266 (49.1)	171 (38.3)	
Undergraduate college/above	195 (12.1)	492 (41.2)		63 (8.9)	76 (10.3)	56 (34.1)		142 (38.5)	180 (33.2)	226 (50.6)	
Professional title			<0.001*				<0.001*				<0.001*
None	1,027 (63.7)	490 (41.0)		448 (63.1)	479 (64.8)	100 (61.0)		110 (29.8)	224 (41.3)	156 (55.1)	
Junior	474 (29.4)	551 (46.1)		222 (31.3)	214 (29.0)	38 (23.2)		204 (55.3)	231 (42.6)	116 (41.0)	
Intermediate/above	112 (6.9)	153 (12.8)		40 (5.6)	46 (6.2)	26 (15.9)		55 (14.9)	87 (16.1)	11 (3.9)	
Number of BPHS undertake			<0.001*				<0.001*				<0.001*
≤2	312 (19.3)	442 (37.0)		118 (16.6)	94 (12.7)	100 (61.0)		106 (28.7)	218 (40.2)	118 (41.7)	
3–4	169 (10.5)	398 (33.3)		90 (12.7)	53 (7.2)	26 (15.9)		157 (42.5)	185 (34.1)	56 (19.8)	
≥5	1,132 (70.2)	354 (29.6)		502 (70.7)	592 (80.1)	38 (23.2)		106 (28.7)	139 (25.6)	109 (38.5)	
Years of TB work			<0.001*				<0.001*				<0.001*
≤2	433 (26.8)	422 (35.3)		137 (19.3)	238 (32.2)	58 (35.4)		67 (18.2)	279 (51.5)	76 (26.9)	
3–4	216 (13.4)	233 (19.5)		88 (12.4)	94 (12.7)	34 (20.7)		67 (18.2)	102 (18.8)	64 (22.6)	
≥5	964 (59.8)	539 (45.1)		485 (68.3)	407 (55.1)	72 (43.9)		235 (63.7)	161 (29.7)	143 (50.5)	
Working satisfaction			0.004*				0.193				0.002*
High satisfaction	799 (49.5)	657 (55.0)		350 (49.3)	357 (48.3)	92 (56.1)		204 (55.3)	274 (50.6)	197 (63.3)	
Low satisfaction	814 (50.5)	537 (45.0)		360 (50.7)	382 (51.7)	72 (43.9)		165 (44.7)	268 (49.4)	104 (36.7)	
Working willingness			<0.001*				0.08				<0.001*
Positive attitude	1,202 (74.5)	784 (65.7)		548 (77.2)	538 (72.8)	116 (70.7)		264 (71.5)	303 (55.9)	217 (76.7)	
Negative attitude	411 (25.5)	410 (34.3)		162 (22.8)	201 (27.2)	48 (29.3)		105 (28.5)	239 (44.1)	66 (23.3)	
TB programme training frequency			<0.001*				<0.001*				<0.001*
≤1/half-year	279 (17.3)	319 (26.7)		48 (6.8)	73 (9.9)	158 (96.3)		39 (10.6)	1 (0.2)	279 (98.6)	
2/half-year	387 (24.0)	454 (38.0)		188 (26.5)	193 (26.1)	6 (3.7)		156 (42.3)	295 (54.4)	3 (1.1)	
≥3/half-year	947 (58.7)	421 (35.3)		474 (66.8)	473 (64.0)	0 (0.0)		174 (47.2)	246 (45.4)	1 (0.4)	

* indicates that *P* < 0.05 via the χ² test.

HCW = health care worker; BPHS = basic public health care services.

Regional, HCWs in Tibet were younger and more educated, while those in Chongqing had longer TB work experience (*P* < 0.001). No regional differences were observed in job satisfaction or willingness among village-level HCWs. At the community level, HCWs in Tibet and Chongqing reported higher job satisfaction and willingness to continue TB work than those in Guizhou (*P* < 0.05). TB training opportunities were more frequent in Chongqing and Guizhou than in Tibet (*P* < 0.001).

**Figure 2.**
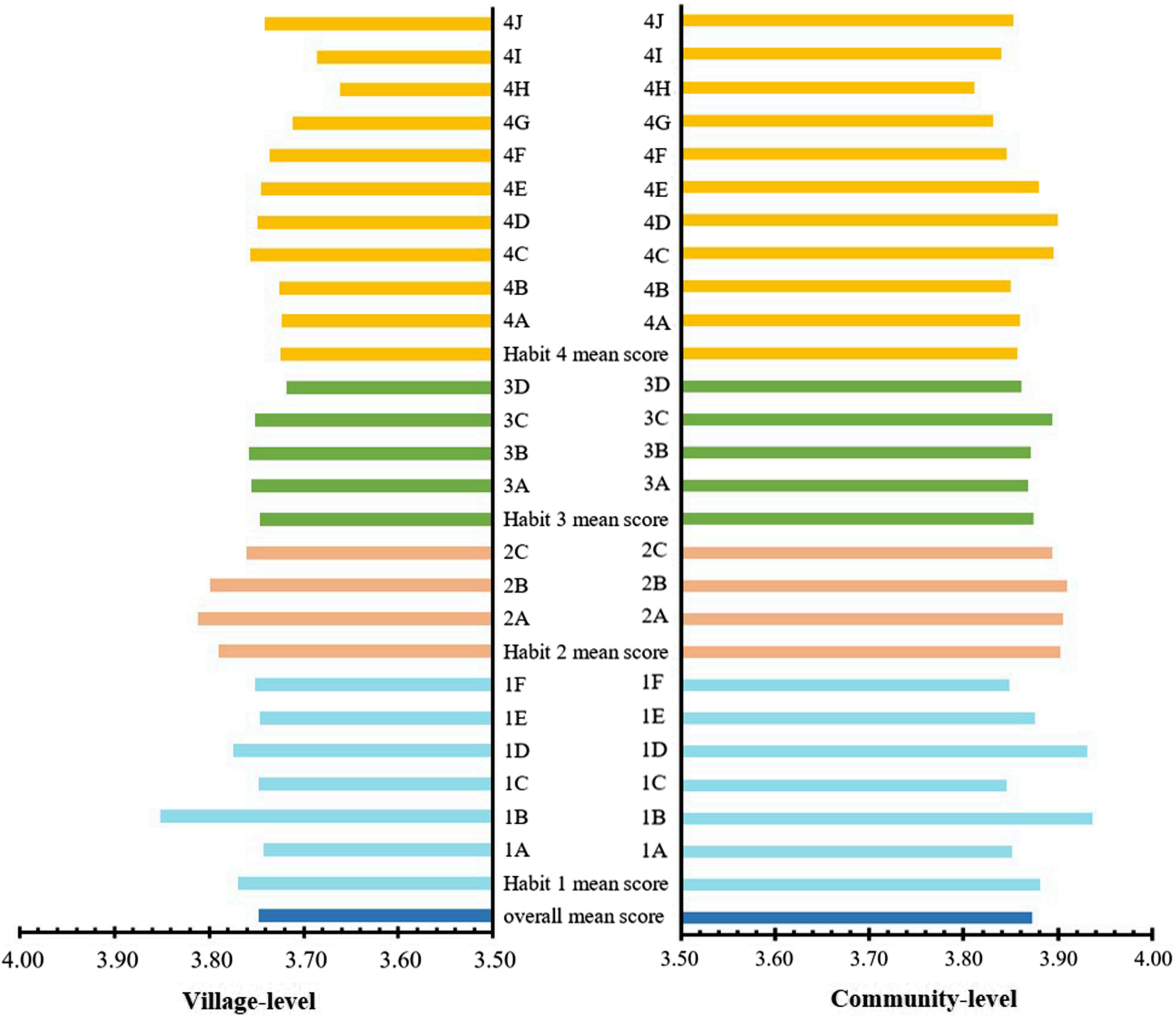
4-HCS mean scores among participated TB HCWs at village level and community level. 1A–1F are part of Habit 1 (Invest in the Beginning); Items 2A–2C are part of Habit 2 (Elicit the Patient’s Perspective); Items 3A–3C are part of Habit 3 (Demonstrate Empathy); Items 4A–4J are part of Habit 4 (Invest in the End). 4-HCS = 4-Habits Coding Scheme; HCW = health care worker.

Among the 2,807 TB HCWs surveyed, the mean 4-HCS score was 3.748(±0.860) for HCWs at the village level and 3.872(±0.842) and for HCWs at the community level (Table 2). At the village level, the mean scores for the four dimensions of the 4-HCS were as follows: Habit 1 (Invest in the Beginning) = 3.769 (±0.922), Habit 2 (Elicit the Patient’s Perspective) = 3.790 (±0.923), Habit 3 (Demonstrate Empathy) = 3.746 (±0.905), and Habit 4 (Invest in the End) = 3.724 (±0.864). At the community level, the corresponding mean scores were 3.881 (±0.876), 3.876 (±0.932), 3.874 (±0.874), and 3.857 (±0.859), respectively. Community-level HCWs achieved significantly higher scores across all dimensions and all 23 items of the 4-HCS compared to village-level HCWs (*P* < 0.05). Across both levels, Habit 2 (Elicit the Patient’s Perspective) had the highest mean scores, while Habit 4 (Invest in the End) had the lowest (Figure 2).

**Table 2.** 4-HCS scores among TB HCWs, mean (SD).

Dimensions	Habit items	Village level (n = 1,613)	Community level (n = 1,194)	*P* value
	Overall mean score	3.748 (0.860)	3.872 (0.842)	<0.001*
Habit 1. Invest in the Beginning	Habit 1 overall mean score	3.769 (0.922)	3.881 (0.876)	0.002*
	1A. Shows familiarity with patient	3.742 (1.093)	3.852 (0.990)	0.029*
	1B. Greets patient warmly	3.851 (1.002)	3.936 (0.945)	0.03*
	1C. Makes small talk	3.748 (1.012)	3.846 (0.970)	0.011*
	1D. Uses primarily open-ended questions	3.774 (1.005)	3.931 (0.916)	<0.001*
	1E. Encourages expansion of patient’s concerns	3.746 (0.999)	3.876 (0.932)	0.001*
	1F. Elicits the full range of concerns	3.751 (0.973)	3.848 (0.923)	0.010*
Habit 2. Elicit the Patient’s Perspective	Habit 2 overall mean score	3.790 (0.923)	3.902 (0.879)	<0.001*
	2A. Interested in patient’s understanding of problem	3.812 (0.969)	3.905 (0.907)	0.013*
	2B. Asks about patient’s goals for visit	3.799 (0.967)	3.909 (0.914)	0.002*
	2C. Shows interest in impact on patient’s life	3.760 (0.955)	3.894 (0.916)	<0.001*
Habit 3. Demonstrate Empathy	Habit 3 overall mean score	3.746 (0.905)	3.874 (0.874)	<0.001*
	3A. Encourages expression of emotion	3.755 (0.962)	3.869 (0.929)	0.001*
	3B. Accepts/validates patient’s feelings	3.758 (0.952)	3.871 (0.917)	0.001*
	3C. Helps to identify/label feelings	3.751 (0.954)	3.894 (0.908)	<0.001*
	3D. Displays effective nonverbal behaviour	3.718 (0.951)	3.861 (0.916)	<0.001*
Habit 4. Invest in the End	Habit 4 overall mean score	3.724 (0.864)	3.857 (0.859)	<0.001*
	4A. Frames information using patient’s perspective	3.723 (0.936)	3.860 (0.916)	<0.001*
	4B. Allows time for information to be absorbed	3.726 (0.938)	3.850 (0.905)	<0.001*
	4C. Explains clearly/uses little jargon	3.757 (0.941)	3.896 (0.905)	<0.001*
	4D. Explains rationale for tests and treatments	3.749 (0.94)	3.900 (0.889)	<0.001*
	4E. Effectively tests for comprehension	3.745 (0.921)	3.880 (0.891)	<0.001*
	4F. Encourages involvement in decision making	3.736 (0.932)	3.846 (0.924)	0.001*
	4G. Explores acceptability of treatment plan	3.711 (0.919)	3.831 (0.919)	<0.001*
	4H. Explores barriers to implementation	3.662 (0.934)	3.812 (0.926)	<0.001*
	4I. Encourages additional questions	3.686 (0.930)	3.840 (0.907)	<0.001*
	4J. Makes clear plans for follow-up	3.741 (0.931)	3.853 (0.916)	0.001*

* indicates that *P* < 0.05 via the Mann–Whitney *U* test.

SD = standard deviation; 4-HCS = 4-Habits Coding Scheme; HCWs = health care workers.

### Factors associated with PCC skill among TB HCWs in PHC settings

Univariate analysis indicated that working willingness, working satisfaction, TB work experience, and PHC institution level were significantly associated with 4-HCS mean scores among the TB HCWs (Supplementary Data). Multiple linear regression analysis revealed that factors associated with higher 4-HCS mean scores among TB HCWs included working willingness, working satisfaction, TB work experience, and PHC institution level (Table 3). TB HCWs with a positive working attitude (*t* = 4.575, *P* < 0.001), high working satisfaction (*t* = 5.559, *P* < 0.001), more years of TB working experience (3–4 years: *t* = 2.721 and *P* = 0.007; ≥5 years: *t* = 4.374 and *P* < 0.001), and those working at community-level PHC settings (*P* < 0.001) had higher 4-HCS mean scores. Among these factors, high working satisfaction and more than 5 years of TB work experience had the greatest impact on explaining the higher 4-HCS scores, as indicated by the highest *t*-values.

**Table 3.** Multiple linear regression analysis of 4-HCS total score among participated TB HCWs.

Variable	Unstandardised coefficient		Standardised coefficient (β)	*t*	*P* value
	β	SE			
Constant	3.566	0.041		87.170	<0.001*
Working willingness (positive attitude)	0.166	0.036	0.088	4.575	<0.001*
Working willingness (negative attitude)	Reference	–	–	–	–
Working satisfaction (high satisfaction)	0.181	0.033	0.106	5.559	<0.001*
Working satisfaction (low satisfaction)	Reference	–	–	–	–
Years of TB work (≥5 years)	0.160	0.037	0.093	4.374	<0.001*
Years of TB work (3–4 years)	0.133	0.049	0.057	2.721	0.007*
Years of TB work (≤2 years)	Reference	–	–	–	–
PHC institution level (village level)	−0.144	0.033	−0.083	−4.429	<0.001*
PHC institution level (community level)	Reference	–	–	–	–

* indicates that *P* < 0.05 via multivariable linear regression analysis.

4-HCS = 4-Habits Coding Scheme; SE = standardised error; HCW = health care worker; PHC = primary health care.

## DISCUSSION

PCC is fundamental to high-quality health care delivery. The 4-HCS is a well-established and extensively validated instrument for assessing PCC across diverse health care settings.^15,23^ In this study, we translated, cross-culturally adapted, and validated the 4-HCS into Chinese and applied it to assess PCC skills among TB HCWs in PHC settings in Western China.

The original US version of the 4-HCS demonstrated acceptable inter-rater reliability and has been widely used for research and training.^23^ Previous cross-cultural adaptations, including the French version, have shown good internal consistency but highlighted the need for rigorous validation procedures to ensure reliability.^15^ A systematic review highlighted that most Chinese instruments assessing patient–doctor communication were self-designed and lacked rigorous validation, underscoring the need for robust tools.^15^ Our findings indicated the Chinese version of the 4-HCS had robust reliability and validity, consistent with prior international adaptations,^16,24^ supporting its suitability for use in Chinese health care settings.

National surveys in China have revealed that inadequate doctor–patient communication has been widely recognised as a major contributor to strained relationships between patients and HCWs.^25,26^ Although communication skills have been assessed using adapted tools such as the Chinese versions of SEGUE framework, with average scores ranging from 14.1 (±3.8) in Nanjing^27^ to 15.1 (±4.0) and 16.0 (±6.3) in Beijing,^28,29^ systematic evaluation using internationally validated instruments remains limited. In this study, TB HCWs demonstrated moderate PCC skills, with community-level HCWs (3.872) scoring higher than village-level HCWs (3.748), and overall scores exceeding those reported in the French 4-HCS study.^15^ Nonetheless, previous research noted that PCC in China was still constrained by short consultation times and limited patient involvement in decision-making, reflecting a lack of patient-centredness.^24^

Community-level TB HCWs consistently outperformed village-level HCWs across all 4-HCS dimensions, likely reflecting differences in educational background, professional capacity, workload, and training opportunities, as reported in earlier studies.^30,31^ Longer TB work experience, higher job satisfaction, and positive work attitudes were significantly associated with better PCC skills, aligning with evidence from both TB and non-TB settings worldwide.^13,32,33^ These findings highlight the importance of stable staffing, supportive work environments, and sustained professional development in enhancing patient-centred care.

Notably, Habit 4 (Invest in the End) received the lowest scores across both PHC levels, suggesting gaps in shared decision-making, treatment planning, and follow-up communication. This finding is consistent with prior studies reporting insufficient psychosocial support and limited PCC in TB care delivery.^31,34^ Targeted, evidence-based training programmes focusing on PCC skills are therefore needed to strengthen patient-centred TB care, particularly in primary care settings.

Our findings have several important implications for improving TB care delivery in PHC settings. Firstly, the consistently lower scores in Habit 4 (‘Invest in the End’) highlight critical gaps in treatment explanation, shared decision-making, and follow-up communication. Targeted training programmes should therefore prioritise strengthening HCWs’ competencies in these areas, particularly in supporting patient understanding of treatment regimens and enhancing adherence counselling. Secondly, the observed disparities between community- and village-level HCWs suggest the need for differentiated capacity-building strategies. Village-level HCWs may benefit from foundational communication skills training, while community-level HCWs may require more advanced training in patient engagement and counselling, alongside structured mentorship mechanisms. Thirdly, given that job satisfaction, work experience, and positive work attitudes were significantly associated with higher PCC scores, system-level interventions – such as improving working conditions, strengthening workforce support, and optimising workload distribution – are essential to sustainably enhance communication quality. Ultimately, improving PCC in TB care has the potential to strengthen treatment adherence, reduce loss to follow-up, and improve overall treatment outcomes, thereby contributing to more effective TB control. Our findings support four key actions for TB programmes: 1) targeted training to address gaps in treatment explanation and shared decision-making; 2) differentiated capacity-building by HCW level; 3) workforce and system-level interventions to improve job satisfaction and working conditions; and 4) strengthened PCC to enhance treatment adherence and reduce loss to follow-up. These additions better position our study as not only a measurement exercise but also a practice- and policy-relevant contribution to strengthening patient-centred TB care. From a TB control perspective, strengthening PCC is critical for improving treatment adherence and reducing loss to follow-up, which are key challenges in TB management. The findings of this study provide actionable evidence for TB programmes in PHC settings. The identified gaps in ‘Invest in the End’ suggest the need to enhance communication around treatment planning and follow-up, which are essential for ensuring continuity of care. In addition, the disparities observed between village- and community-level HCWs support the implementation of differentiated training and supervision strategies within TB programmes. Furthermore, the association between job satisfaction and PCC performance highlights the importance of workforce support and system-level interventions in improving the quality of TB care. Collectively, these findings offer practical guidance for integrating PCC into TB control strategies and strengthening the effectiveness of PHC-based TB services.

This study has several limitations. It was conducted exclusively among TB HCWs in Western China, which may limit generalisability. In addition, reliance on self-reported data may introduce social desirability bias. Future studies should incorporate objective assessments, such as direct observation or patient-reported experiences, and include broader geographic regions.

In this study, the Chinese version of the 4-HCS was administered as a self-reported instrument, which differs from the original observer-rated application of the tool. Although this approach enabled large-scale data collection across geographically diverse and resource-constrained PHC settings, it may introduce social desirability and reporting bias, potentially leading to overestimation of PCC skills. Therefore, the findings should be interpreted as reflecting HCWs’ self-perceived PCC competencies rather than objectively assessed communication behaviours. Furthermore, the lack of external observational validation limits direct comparability with prior international studies that employed independent raters to code recorded clinical encounters. Future research should incorporate objective assessment methods, such as audio- or video-recorded consultations evaluated by trained raters, to further validate the Chinese 4-HCS and strengthen its applicability across settings.

## CONCLUSIONS

The Chinese version of the 4-HCS is a reliable and valid instrument for assessing PCC among HCWs. Application of this tool revealed moderate PCC skills among TB HCWs in Western China, with notable disparities by PHC level and work characteristics. Strengthening PCC training, improving job satisfaction, and supporting experienced HCWs may enhance patient-centred TB care and contribute to better treatment outcomes.
